# Identification of the *CDPK* gene family in patchouli and functional analysis in response to continuous cropping stress

**DOI:** 10.3389/fpls.2023.1300073

**Published:** 2023-11-22

**Authors:** Xiaofeng Liu, Muhammad Zeeshan Ul Haq, Jing Yu, Ya Liu, Huageng Yang, Hongguang Cui, Dongmei Yang, Yougen Wu

**Affiliations:** School of Breeding and Multiplication (Sanya Institute of Breeding and Multiplication), School of Tropical Agriculture and Forestry, Hainan University, Sanya, China

**Keywords:** *Pogostemon cablin* (patchouli), *CDPK* gene family, continuous cropping disorders, gene cloning, subcellular localization

## Abstract

To further reveal the molecular mechanisms underlying the formation of continuous cropping disorders in patchouli, this study analyzed the function of calcium dependent protein kinase (*CDPK*) genes at the molecular level in patchouli continuous cropping disorders. The findings unveiled the presence of 45 *PcCDPK* genes within the complete patchouli genome dataset. These genes exhibited a range of molecular weights from 50.78 to 78.96 kDa and aliphatic index values spanning from 74.42 to 88.49, and are shown to be hydrophilic proteins. The evolution of 45 *PcCDPK* members was divided into 4 subfamilies, with a total of 65 pairs of collinear genes. Each PcCDPK contains a STKc-CAMK domain and four EF-hand structures exhibiting a certain degree of conservatism during evolution. Transcriptome data further supported the significance of *PcCDPK25* and *PcCDPK38* genes, showing substantial upregulation, which was corroborated by qRT-PCR results. The 1629 bp and 1716 bp CDS sequences were obtained by cloning the *PcCDPK25* and *PcCDPK38* genes, respectively, and subcellular localization showed that both proteins were localized on the plasma membrane. This comprehensive study provides molecular-level confirmation of the pivotal roles played by *CDPK* genes in the emergence of continuous cropping challenges in patchouli plants, establishing a crucial foundation for a deeper comprehension of the molecular mechanisms underpinning these obstacles.

## 1 Introduction

Patchouli (*Pogostemon cablin* Blanco Benth.), a medicinal plant belonging to the *Lamiaceae* family, originates from Southeast Asian nations like the Philippines, Malaysia, and Vietnam (Maheshwari et al., 1993). Its primary cultivation zones are in the southern regions of China, specifically Hainan and Guangdong (Wu et al., 2010). Patchouli is used as a medicinal herb with its dry aboveground parts, and is commonly used for pregnancy vomiting, pretreatment of influenza, anti-inflammatory and analgesic effects, indigestion, headache and fever, etc. (Chakrapani et al., 2013). In addition, *P. cablin* essential oil extracted from patchouli plants contains abundant patchouli alcohol (PA) and patchouli ketone (PO), which are important ingredients in the perfume industry to provide durability for perfume (Zhang et al., 2018). From this, it can be seen that patchouli has important pharmaceutical and industrial value worldwide, and has broad application prospects.

In the traditional Chinese medicine market, patchouli is categorized into four distinct varieties based on its cultivation regions: Shipai patchouli (“Paixiang”), Zhaoqing patchouli (“Zhaoxiang”), Zhanjiang patchouli (“Zhanxiang”), and Hainan patchouli (“Nanxiang”) (Wu et al., 2010). However, there are serious continuous cropping obstacles in the cultivation of patchouli, leading to the degradation, browning, and decay of the underground roots of patchouli, weak growth of aboveground leaves, reduced branching, poor plant growth, and even whole plant death, which has become a bottleneck problem that restricts the quality and sustainable development of patchouli medicinal materials (Zeng et al., 2020; Zeeshan Ul Haq et al., 2023). Allelopathic autotoxicity is one of the important reasons affecting continuous cropping disorders in patchouli plants (Bi et al., 2010; Ren et al., 2015). The isolated 45 allelopathic substances from the rhizosphere of patchouli, among which p-HBA (p-hydroxybenzoic acid) had the strongest allelopathic and self-toxic effect on patchouli tissue cultured seedlings (Xu et al., 2015). When studying patchouli seedlings exposed to varying concentrations of p-HBA, it was observed that the pivotal threshold for stress activation was at a concentration of 1mmol/L p-HBA. This concentration notably upregulated the expression of genes associated with *CDPK*, Ca^2+^/calmodulin-dependent EF-Hand protein kinase, ethanol dehydrogenase, and other relevant genes (Yan et al., 2022). Furthermore, during the analysis of gene expression disparities between continuous cropping and first cropping, notable activation was observed in *CDPK* genes within the calcium signaling pathway and MAPK genes within the pathway (Yan et al., 2023).

Plants react to diverse biotic and abiotic stresses by elevating the intracellular concentration of Ca^2+^, a widely prevalent second messenger (Tang and Luan, 2017). In plants, there are three main families of Ca^2+^ sensors: calmodulin B-like proteins (CBLs), CaM/calmodulin like proteins (CMLs), and calcium-dependent protein kinases CDPKs (Boudsocq and Sheen, 2013). Unlike other Ca^2+^ sensors, CDPKs have the dual functions of Ca^2+^ sensors and responders due to their unique structural characteristics, allowing them to directly perceive and respond to calcium signals and convert them into downstream protein phosphorylation events (Poovaiah et al., 2013). Research has found that in Arabidopsis, plants overexpressing *AtCPK6* and *AtCPK3* exhibit increased tolerance to salt and drought stress, and the *AtCPK21* and *AtCPK23* mutants exhibit increased tolerance to high osmotic, drought, and salt stress, while the *AtCPK6* mutant plants do not exhibit significant phenotypic expression (Mori et al., 2006; Mehlmer et al., 2010). *AtCPK8* regulates Arabidopsis response to drought stress through phosphorylation of CAT3 (Zou et al., 2015). In rice, overexpression of *OsCDPK7* genes has been shown to enhance the resistance of rice to cold, drought, and salt stress (Saijo et al., 2010). Overexpression of *OsCDPK12* increases tolerance to salt stress and sensitivity to compatible and incompatible rice blast fungi (Asano et al., 2012a). *OsCDPK9* plays a special role in the signal transduction response of rice blast disease (Wei et al., 2014). Numerous studies have reported that the *CDPK* genes play a vital role in response of plants to abiotic and biotic stresses (Dontoro et al., 2022; Li et al., 2022). The 14 and 17 *CDPK* genes were identified in *Prunus mume* and *Prunus mume* (var. Tortuosa) genomes, respectively, and their roles in cold stress response were pointed out (Miao et al., 2023). The *CDPK* genes have also been discovered in transcriptome data of wheat response to drought stress, and the expression of *TaCDPK25* has been proved to be positively regulated by *TaDREB3* to improve plant drought tolerance *in vivo* and *in vitro* (Bin et al., 2023). The 21 *CDPK* key genes were found to be involved in the continuous cropping of specific signal transduction pathways in *Rehmannia glutinosa*. *RgCDPK10* is homologous to *PcCDPK25*, and *RgCDPK18* is homologous to *PcCDPK38*. Therefore, *PcCDPK* genes, especially *PcCDPK25* and *PcCDPK38*, are likely involved in the signal response process of patchouli to continuous cropping disorders (Yang et al., 2013; Yang et al., 2017a). Studying their structure and function is of great significance for exploring the regulatory mechanism of genes in the continuous cropping of patchouli in the future.

This study excavated *CDPKs* from the transcriptome library of patchouli continuous cropping in the early stage of the research group, and identified 45 *CDPK* gene family members from the entire patchouli genome. The phylogenetic relationships and gene structures among gene members were studied, and the *PcCDPK25* and *PcCDPK38* genes, which are highly likely to regulate continuous cropping disorders in patchouli, were cloned and subcellular mapped. The expression patterns of these two genes under continuous cropping stress and p-HBA stress were analyzed using real-time fluorescence quantitative qRT-PCR. This study provides important technical support for revealing the mechanism of *CDPKs* in the formation of continuous cropping disorders in *P. cablin* and improving the key molecular mechanisms of continuous cropping disorders.

## 2 Materials and methods

### 2.1 Identification of *PcCDPK* gene family members and analysis of protein physicochemical properties

Download the gene sequences of Arabidopsis *CDPK* members from the Arabidopsis genome database (https://www.arabidopsis.org/) and compare them with the patchouli genome data (PRJNA647235) (Shen et al., 2022). The obtained sequences were further screened using the hidden Markov model of CDPK’s EF-hand domain (EF-hand, Pfam ID: PF00036) and serine/threonine protein kinase (Ser-TKc, Pfam ID: PF07714) to obtain 45 members of the patchouli *CDPK* gene family (Shi and Zhu, 2022). The physicochemical characteristics of PcCDPK family proteins, including amino acid count (aa), molecular weight (MW), isoelectric point (pI), and protein hydrophilicity (GRAVY), were assessed using the Expasy database (https://web.expasy.org/protparam/) (Duvaud et al., 2021).

### 2.2 Evolutionary tree construction and chromosomal collinearity analysis of the *PcCDPK* gene family

31 OsCDPKs obtained from the rice (*Oryza sativa* L.) database (https://riceome.hzau.edu.cn/) were compared with 34 AtCDPKs and 45 PcCDPKs using TBtools and MEGA-X software to analyze phylogenetic relationships (Jones et al., 1992; Kumar et al., 2016). Furthermore, according to the annotated genome file of patchouli, the chromosome position information and collinearity relationships of *PcCDPK* gene family members were obtained (He et al., 2022a).

### 2.3 Conservative motif and gene structure analysis of *PcCDPK* gene family proteins

In order to study the gene structure of *PcCDPK* family members, based on the CDPK protein sequence of patchouli, the gene structure and phylogenetic evolution of PcCDPK were obtained through TBtools and MEGA-X software. The MEME online software (https://meme-suite.org/meme/) was used to predict the protein conserved motifs of *PcCDPK*. Finally, the gene structure, phylogenetic evolution, and conserved motifs were visualized as graphs (Bailey et al., 2015; Chen et al., 2020).

### 2.4 Analysis of cis-acting elements of promoters

Using TBtools software to extract 2000 bp DNA sequence of the starting codon (ATG) of *PcCDPK* gene family members from annotation files, using Plant-Care (https://bioinformatics.psb.ugent.be/webtools/plantcare/html/) to predict cis-acting elements, and then using Excel to organize and plot (Lescot et al., 2002).

### 2.5 Expression analysis of *PcCDPKs* in transcriptome data

To investigate the functional distinctions among members of the *CDPK* gene family in patchouli, an analysis was conducted using transcriptome data obtained from previous research involving continuous cropping and p-HBA stress experiments on patchouli (PRJNA737065, PRJNA850618) (Yan et al., 2022; Yan et al., 2023). This analysis focused on examining the expression patterns of *CDPK* gene family members in both the roots and leaves of patchouli subjected to varying durations of continuous cropping. Additionally, it assessed the expression profiles of these genes in the roots of patchouli seedlings exposed to 1mmol/L p-HBA stress. The results were visualized through the creation of heat maps.

### 2.6 Gene cloning and sequence analysis of *PcCDPK25* and *PcCDPK38*

The cDNA extracted from the root system of patchouli served as the template for PCR amplification of the *PcCDPK25* and *PcCDPK38* genes. Specific primers, designed using Primer 5.0 software, were employed for this purpose (**Table 1**). The PCR amplification was carried out in a 25 μL reaction system, comprising 0.5 μL of template DNA, 0.5 μL of Primer F/R, 12.5 μL of Prime STAR Max (TAKARA), and 11 μL of ddH_2_O. The resulting PCR products were subjected to electrophoresis on a 1% agarose gel for detection and subsequent recovery. The recovered cDNA was then integrated into the pBM16A vector using a Topomart cloning kit, followed by the transformation of the recombinant plasmid into *E. coli*. Positive single colonies were selected for bacterial solution PCR, and the correctly identified bacterial solution underwent overnight shaking for 12-16 hours before being sent for sequencing. Upon receiving the sequencing results, a comparative analysis was conducted against the gene sequences of *PcCDPK25* and *PcCDPK38*, with further examination of protein structure and evolutionary traits.

**Table 1:** Cloning and qRT-PCR primers of *PcCDPK25*、*PcCDPK38*.

Gene name	Application	Primer F	Primer R
*PcCDPK25*	Clone	ATGGGAAATTGCAACGCCT	TCAAACCATAACAGTGTGACCAG
*PcCDPK38*		ATGGGCAGCTGTTTTTCTACCA	TCAGTGCAGCGGCATCTTCT
*PcCDPK25*	qRT-PCR	CCTGGTGAAAGGTTCTCTGA	ACACCTTGTTCCGTTTCAGC
*PcCDPK38*		CGTTTTTCGTCTGCTCGTAT	TTGAGGGCTACGATGAACAC

### 2.7 Subcellular localization

Recombinant plasmids pBM16A-*PcCDPK25* and pBM16A-*PcCDPK38* were used as templates for homologous arm primer amplification. The amplified fragments were recovered and connected to the linearized vector pBWA (V) HS-GFP digested by BsaI and Eco31I using the homologous recombination method to obtain recombinant plasmids pBWA (V) HS-*PcCDPK25*-GFP and pBWA (V) HS-*PcCDPK38*-GFP. Two recombinant vectors were transfected into *Agrobacterium tumefaciens* GV3101 host cells, followed by propagation. Subsequently, these transformed cells were introduced into the lower epidermis of indigenous tobacco leaves. After a 2-day exposure to light, their localization was examined using a confocal microscope.

### 2.8 Quantitative real-time PCR analysis

Root RNA was extracted from patchouli at the S2, S3, and S4 growth stages during the initial crop and after continuous cropping for one year. Leaf RNA was obtained from patchouli at the S2 and S4 stages in the first crop, continuous cropping for one year, and two years. Additionally, root RNA was collected from patchouli seedlings subjected to 1mM p-HBA stress for varying durations (0 h, 6 h, 12 h, 24 h, 48 h, and 96 h). The resulting cDNA, synthesized through reverse transcription, served as the template for quantitative real-time PCR. In this qRT-PCR process, 18S ribosomal RNA (18S rRNA) was employed as the internal reference gene. The reaction system consisted of 20 μL, comprising MonAmp™ ChemoHS qPCR Mix (10 μL), Primer F (0.4 μL), Primer R (0.4 μL), cDNA (0.5 μL), and ddH_2_O (8.7 μL). The expression levels of the *PcCDPK25* and *PcCDPK38* genes were assessed using quantitative real-time PCR, and the analysis was conducted using the 2^-ΔΔCt^ method for calculation.

## 3 Results

### 3.1 Identification of *PcCDPK* gene family members and analysis of protein physicochemical properties

Using hidden Markov models (Pfam: PF00036, PF07714) and local blast of the Arabidopsis *CDPK* gene family, 45 *PcCDPK* gene family members were identified from patchouli genome data and named *PcCDPK1*~*PcCDPK45* (**Supplementary Table S1**). According to the analysis of protein physicochemical properties (**Table 2**), the length of the protein encoded by the *PcCDPK* genes ranges from 445 (PcCDPK35) to 694 (PcCDPK10) amino acids (aa), and molecular weight (MW) ranges from 50.78 kDa (PcCDPK35) to 78.96 kDa (PcCDPK10). Except for PcCDPK38, PcCDPK39, PcCDPK41, PcCDPK42, and PcCDPK44 proteins with isoelectric points (pI) greater than 9, the other 40 members’ coding proteins have isoelectric points (pI) between 5.19 (PcCDPK15) and 6.57 (PcCDPK25), indicating that most PcCDPKs proteins are acidic. The instability coefficient (II) of the protein ranges from 32.06 to 49.45, with 20 proteins having an II<40 indicating good protein stability, and 25 proteins having an II>40 indicating that the protein may be unstable. The fat coefficient (AI) ranges from 74.42 (PcCDPK18) to 88.49 (PcCDPK34), and the overall average range of the hydrophilicity index (GRAVY) is -0.298 (PcCDPK2) to -0.625 (PcCDPK1), indicating that PcCDPK is a hydrophilic protein. The instability coefficient (II) of the protein ranges from 32.06 to 49.45, with 20 proteins having an II<40 indicating good protein stability, and 25 proteins having an II>40 indicating that the protein may be unstable. The fat coefficient (AI) ranges from 74.42 (PcCDPK18) to 88.49 (PcCDPK34), and the overall average range of the hydrophilicity index (GRAVY) is -0.298 (PcCDPK2) to -0.625 (PcCDPK1), indicating that PcCDPK is a hydrophilic protein. Except for a few members such as the PcCDPK4 protein located in the peroxisome and the PcCDPK7 protein located in the nucleus, most PcCDPK protein subcellular localization is predicted in the cytoskeleton or chloroplast, indicating that most proteins’ function in the cytoskeleton and chloroplast.

**Table 2:** Physical and chemical properties of *PcCDPK* gene family protein.

Gene name	Gene ID	No. of aa	MW (kDa)	pI	II	AI	GRAVY	Subcellular localization
*PcCDPK1*	Pat_A04G087900.m1	545	62.10	6.44	41.21	74.61	-0.625	Cytoskeleton
*PcCDPK2*	Pat_A14G046000.m1	491	55.37	5.81	32.50	84.15	-0.298	Cytoskeleton
*PcCDPK3*	Pat_A13G045200.m1	595	67.34	5.68	38.16	78.77	-0.493	Cytoskeleton
*PcCDPK4*	Pat_B03G222700.m1	512	57.05	5.93	38.90	80.18	-0.389	Peroxisome
*PcCDPK5*	Pat_B17G006800.m1	602	66.92	5.33	45.18	79.87	-0.441	Chloroplast
*PcCDPK6*	Pat_A09G064200.m1	519	58.99	5.60	47.46	81.16	-0.482	Cytoskeleton
*PcCDPK7*	Pat_B13G042800.m1	614	69.29	5.56	42.32	77.65	-0.440	Nucleus
*PcCDPK8*	Pat_B06G063300.m1	542	61.79	6.54	37.27	82.90	-0.445	Cytoskeleton
*PcCDPK9*	Pat_B13G041700.m1	580	64.87	5.25	44.47	82.50	-0.407	Chloroplast
*PcCDPK10*	Pat_B17G008400.m1	694	78.96	5.90	49.45	79.70	-0.521	Cytoskeleton
*PcCDPK11*	Pat_A05G198900.m1	517	57.70	6.01	34.49	83.97	-0.352	Cytoskeleton
*PcCDPK12*	Pat_A17G007600.m1	652	73.77	5.57	49.34	80.49	-0.475	Cytoskeleton
*PcCDPK13*	Pat_B14G041200.m1	582	65.11	5.26	44.30	83.40	-0.401	Chloroplast
*PcCDPK14*	Pat_A23G044900.m1	578	64.35	5.28	37.17	80.28	-0.384	Chloroplast
*PcCDPK15*	Pat_B18G122800.m1	523	58.15	5.19	41.92	75.22	-0.484	Cytoskeleton
*PcCDPK16*	Pat_B05G063200.m1	542	61.72	6.51	36.45	82.90	-0.435	Cytoskeleton
*PcCDPK17*	Pat_B27G064700.m1	536	59.71	5.87	34.77	77.00	-0.453	Chloroplast
*PcCDPK18*	Pat_A18G143800.m1	530	58.89	5.20	42.38	74.42	-0.503	Cytoskeleton
*PcCDPK19*	Pat_B28G057000.m1	536	59.69	5.87	34.79	77.18	-0.443	Chloroplast
*PcCDPK20*	Pat_B17G128600.m1	533	59.12	5.20	41.54	74.73	-0.490	Cytoskeleton
*PcCDPK21*	Pat_B21G142900.m1	517	58.16	5.59	37.44	86.00	-0.333	Nucleus
*PcCDPK22*	Pat_A22G157300.m1	515	57.97	5.40	37.60	86.91	-0.327	Nucleus
*PcCDPK23*	Pat_B03G169500.m1	566	63.12	5.66	40.13	82.54	-0.357	Cytoskeleton
*PcCDPK24*	Pat_B18G008000.m1	619	70.03	5.59	44.25	80.55	-0.449	Cytoskeleton
PcCDPK25*	Pat_B26G112800.m1	542	61.50	6.57	33.75	83.62	-0.427	Cytoskeleton
*PcCDPK26*	Pat_A18G006800.m1	592	65.75	5.25	45.18	80.56	-0.435	Cytoskeleton
*PcCDPK27*	Pat_B18G006500.m1	596	66.30	5.47	44.72	80.82	-0.421	Chloroplast
*PcCDPK28*	Pat_A18G008400.m1	661	74.83	5.60	47.08	80.71	-0.483	Nucleus
*PcCDPK29*	Pat_B09G015700.m1	499	56.37	5.32	42.68	86.15	-0.324	Chloroplast
*PcCDPK30*	Pat_A14G044800.m1	613	68.53	5.23	42.88	85.84	-0.342	Chloroplast
*PcCDPK31*	Pat_A31G140000.m1	531	60.17	5.89	32.06	80.94	-0.488	Chloroplast
*PcCDPK32*	Pat_A13G020300.m1	528	59.67	5.83	38.11	86.59	-0.406	Chloroplast
*PcCDPK33*	Pat_B20G061000.m1	537	61.60	5.39	38.00	87.49	-0.454	Plasma membrane
*PcCDPK34*	Pat_A20G064200.m1	543	62.15	5.23	38.52	88.49	-0.428	Peroxisome
*PcCDPK35*	Pat_B21G061900.m1	445	50.78	6.02	42.63	85.89	-0.422	Cytoskeleton
*PcCDPK36*	Pat_B28G048400.m1	531	59.87	6.35	35.11	81.34	-0.472	Endoplasmic reticulum
*PcCDPK37*	Pat_B19G063100.m1	548	62.82	5.36	37.35	87.68	-0.445	Plasma membrane
PcCDPK38*	Pat_A21G067600.m1	571	64.32	9.11	42.65	77.37	-0.568	Chloroplast
*PcCDPK39*	Pat_B22G054400.m1	571	64.37	9.17	44.72	77.02	-0.573	Chloroplast
*PcCDPK40*	Pat_A17G023400.m1	528	59.62	5.84	38.18	83.83	-0.419	Chloroplast
*PcCDPK41*	Pat_B28G104000.m1	562	63.67	9.11	40.84	77.92	-0.590	Chloroplast
*PcCDPK42*	Pat_A22G056600.m1	571	64.39	9.11	46.11	77.02	-0.576	Chloroplast
*PcCDPK43*	Pat_A27G025000.m1	531	60.00	6.46	36.95	81.88	-0.479	Endoplasmic reticulum
*PcCDPK44*	Pat_A27G084500.m1	562	63.60	9.16	40.61	76.89	-0.592	Chloroplast
*PcCDPK45*	Pat_A04G006200.m1	529	59.88	5.88	40.64	80.53	-0.501	Cytoskeleton

### 3.2 Phylogenetic and chromosomal collinearity analysis of PcCDPK proteins

**Figure 1:**
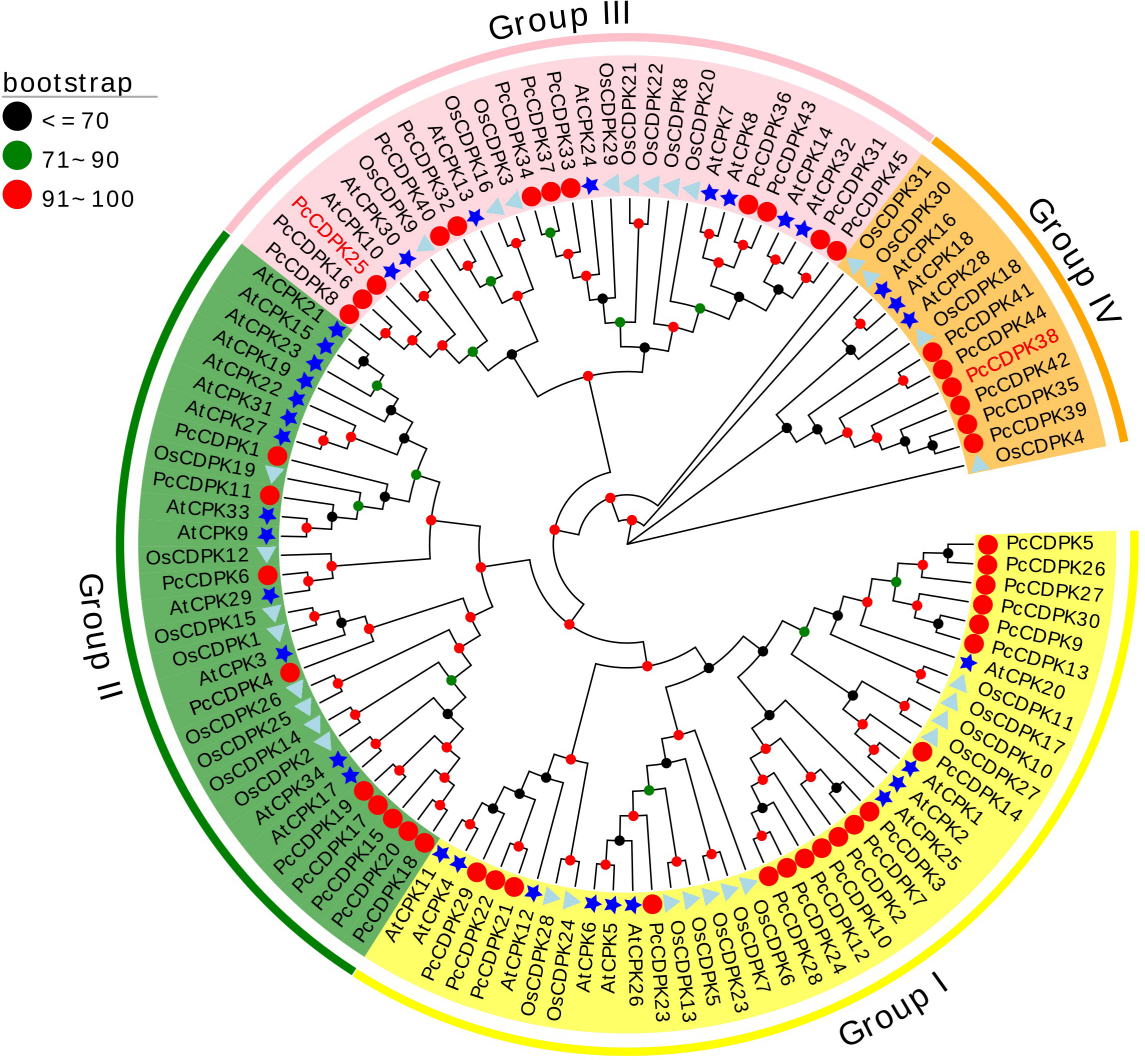
Phylogenetic relationships between CDPK protein members in patchouli (red circle), Arabidopsis (blue star), and rice (cyan triangle). The bootstrap number displays the branch reliability expressed as a percentage. Yellow, green, pink, and orange represent the four phylogenetic groups I, II, III, and IV of CDPKs, respectively.

In order to study the evolutionary relationship between the CDPK gene family proteins in Arabidopsis, rice, and patchouli, a phylogenetic tree was constructed using the reported protein sequences of 34 AtCPKs, 31 OsCDPKs, and 45 PcCDPKs. Based on the classification of Arabidopsis CDPKs proteins, the members of the patchouli CDPK family were separated into 4 subfamilies: Group I, II, III, and IV, each containing 18, 9, 12, and 6 PcCDPK family members respectively. Compared to rice, the evolutionary relationship between patchouli CDPKs and Arabidopsis CDPKs is closer, such as AtCPK29 and PcCDPK6 in the same evolutionary branch, indicating that the two may have similar functions (**Figure 1**).

**Figure 2:**
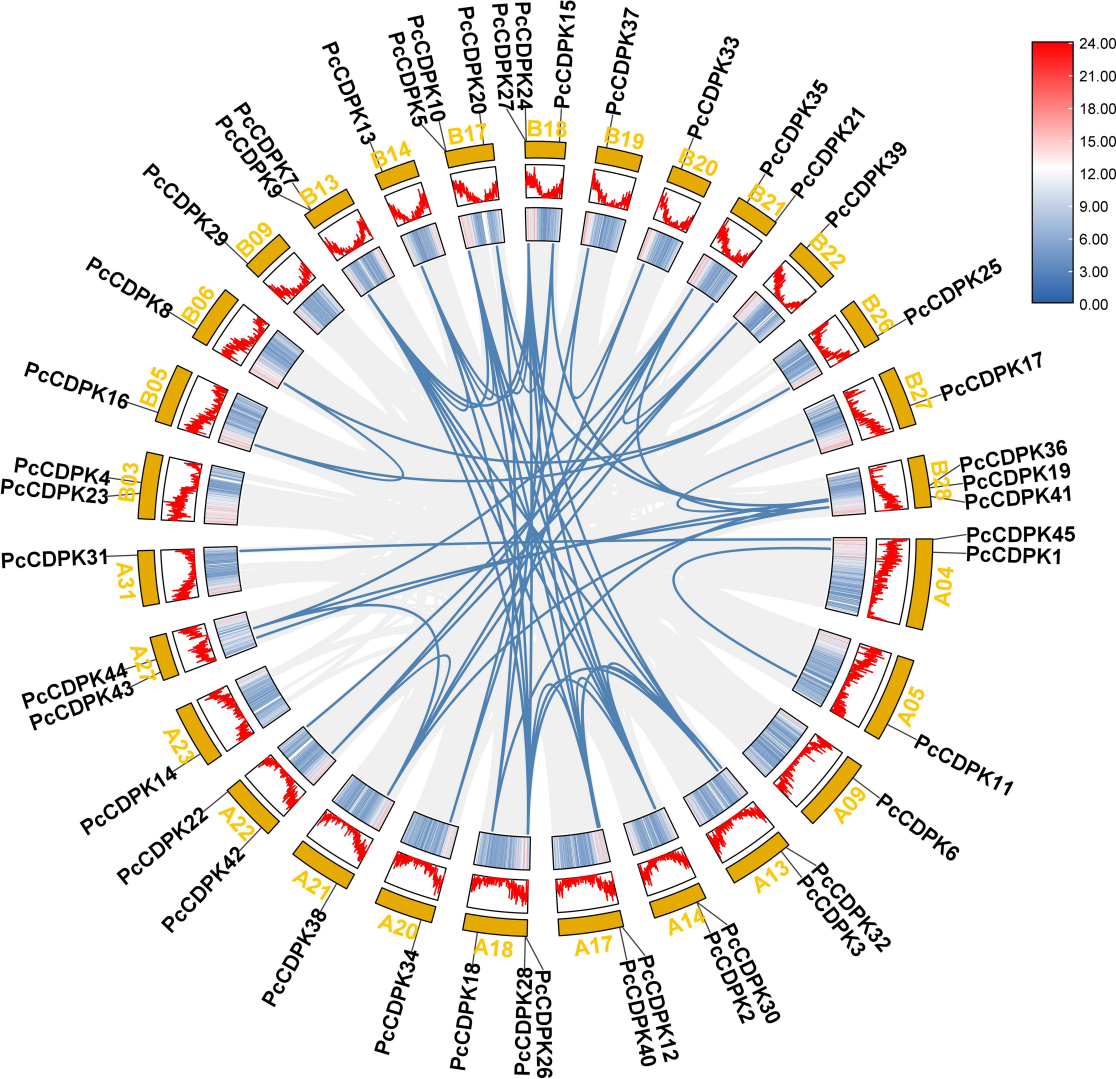
The position and collinearity relationship of *PcCDPKs* members on chromosomes. The yellow color block represents the chromosome, and the corresponding gradient icon and peak map represent the gene density on the chromosome.

45 *PcCDPK* genes were identified in the entire genome data of patchouli, distributed on 28 of the 64 chromosomes of patchouli (**Figure 2**). Based on the annotation information of the genome, the position of genes on chromosomes and the collinearity relationships between family members are obtained. As shown in **Figure 2**, each of the 28 chromosomes contains 1-3 *PcCDPK* genes, among which chromosomes A18, B17, B18, and B28 each have 3 *PcCDPK* genes. A total of 65 pairs of collinearity genes were found among 45 *PcCDPK* genes, such as *PcCDPK33*/*PcCDPK34*, *PcCDPK8*/*PcCDPK16*, *PcCDPK31*/*PcCDPK45*, etc., indicating that they belong to fragment duplication and may be homologous genes capable of performing the same function.

### 3.3 Conservative motifs and gene structure analysis of the *PcCDPK* Gene family

**Figure 3:**
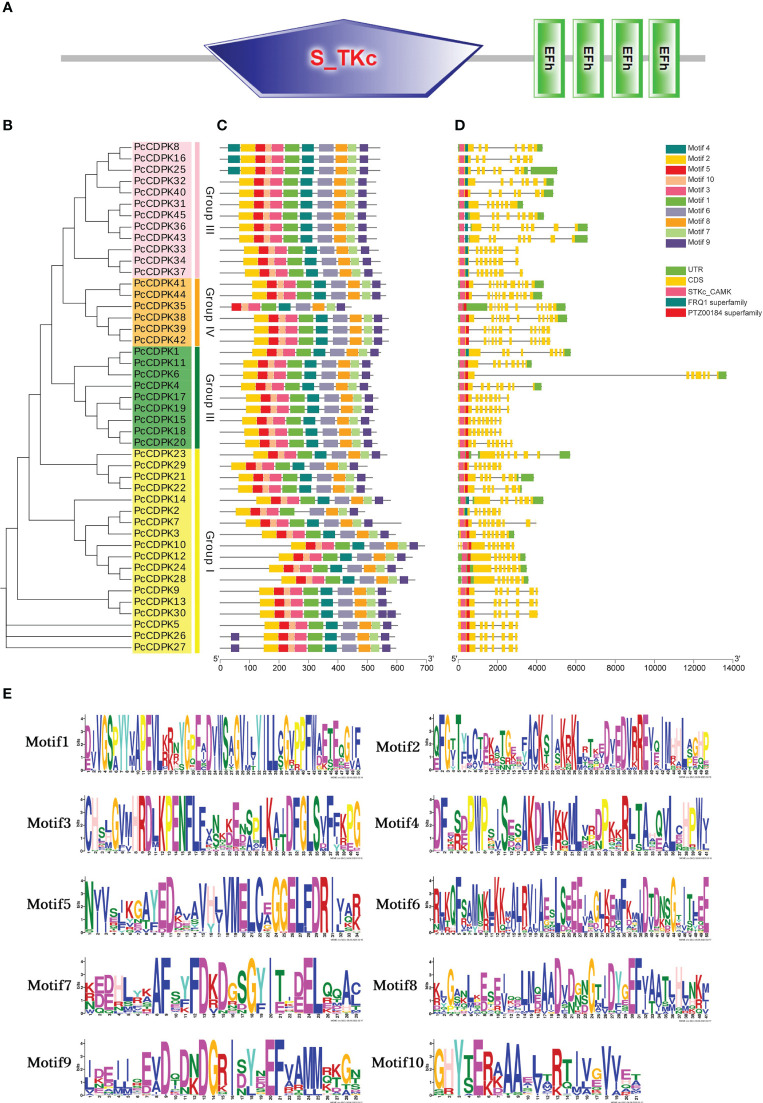
Gene structure and conserved motif of PcCDPK. **(A)** Domain prediction of 45 PcCDPK proteins. **(B)** Phylogenetic tree of 45 PcCDPK proteins **(C)** Conservative motifs of PcCDPK proteins **(D)** Gene structure of *PcCDPK* genes **(E)** Sequence identification of 10 conserved motifs.

To investigate the sequence information and structural characteristics of the *PcCDPK* genes in patchouli, we analyzed the gene structures (**Figures 3A-D**) and conserved motifs (**Figure 3C**) of 45 gene family members, and the sequences of 10 motifs are shown in **Figure 3E**. Gene structure analysis shows that the gene structure of *PcCDPK* has high similarity, with 45 PcCDPKs containing a serine/threonine-like protein kinase domain (STKc_CAMK) and 4-EF hand structures, which enable them to respond to calcium signal transduction and undergo phosphorylation reactions at specific sites of serine and threonine residues. The coding sequence (CDS) of the *PcCDPK* genes is 7-12, and the number of untranslated regions (UTRs) is 0-3. Combining evolutionary clustering, it was found that members of the same subfamily of PcCDPK exhibit similar gene structures and protein conserved motif distributions. For instance, the Group III subfamily contains the FRQ1 superfamily except for PcCDPK40, and the Group IV subfamily contains the PTZ00184 superfamily.

The members of the *PcCDPK* gene family have high consistency in conserved motifs. Except for the absence of Motif4 in PcCDPK2 and Motif2 in PcCDPK35, all 43 PcCDPKs contain Motif1 Motif10. PcCDPK8, PcCDPK16, and PcCDPK25 contain two Motif4, while PcCDPK26 and PcCDPK27 also contain two Motif9, located at the beginning and end of the gene. Although the motif composition varies among different subfamilies, the same type of CDPK protein typically exhibits similar motifs. In summary, the key sequences of the *PcCDPK* genes structure still exhibit a certain degree of conservation during evolution.

### 3.4 Analysis of cis-acting elements in *PcCDPKs* promoter

**Figure 4:**
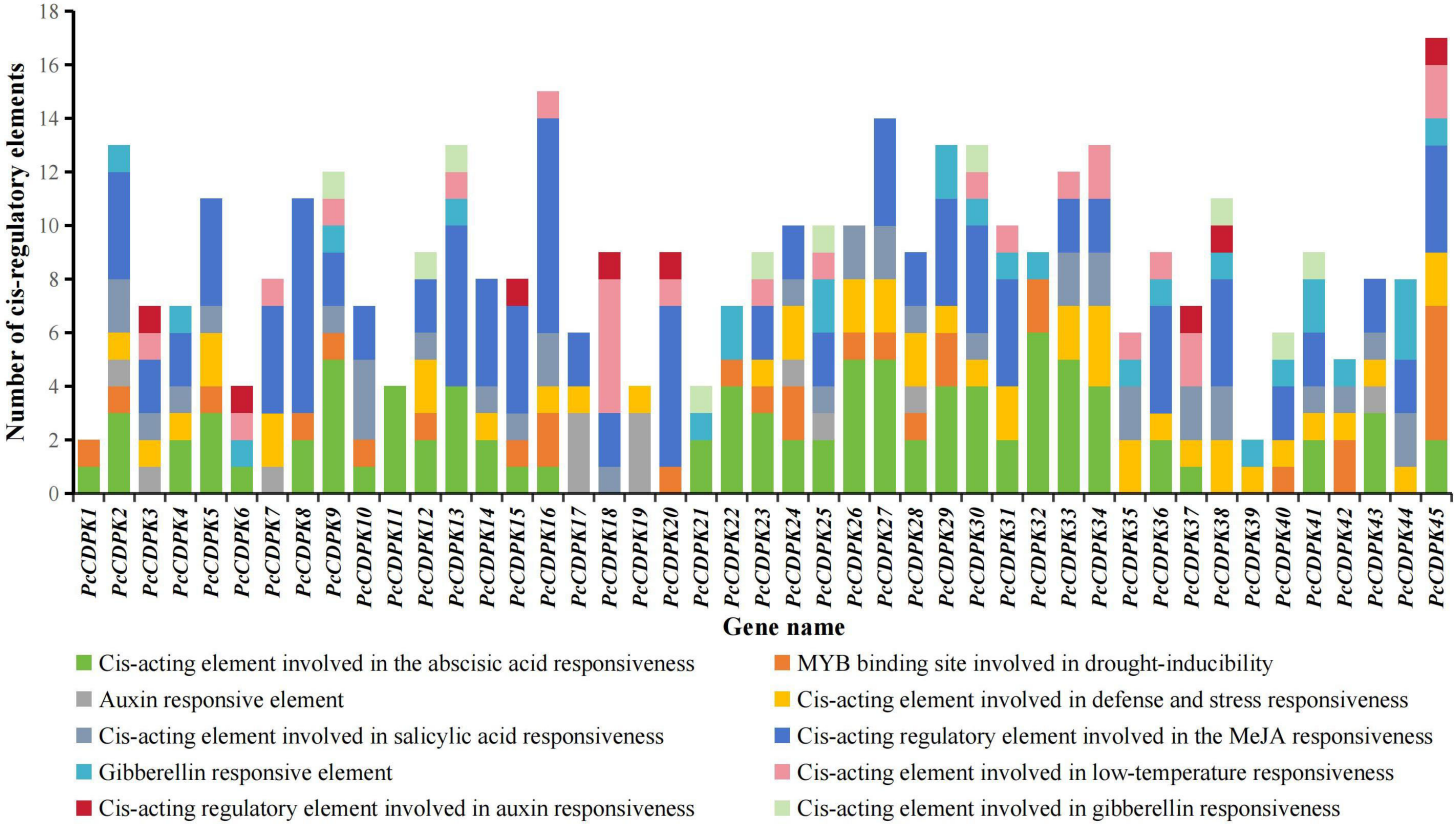
Number of cis-regulatory elements in the *PcCDPKs* promoter region.

There is a certain correlation between the cis-acting elements in the promoter region and gene specific response expression. The PlantCARE online tool was used to analyze the cis-acting elements of the promoters of patchouli *CDPK* genes, and it was found that the *PcCDPK* genes promoter sequences contain multiple cis-acting elements that respond to stress and plant hormones (**Figure 4**). The promoter regions of all *CDPK* genes contain at least one hormone responsive element, such as Abscisic acid (ABA) responsive element ABRE, Methyl jasmonate (MeJA) responsive element TGACG-motif, Salicylic acid (SA) responsive element TCA-element, Auxin (IAA) responsive element TGA-element, and AuxRR-core. Among them, the promoter regions of most *CDPK* genes were found to respond to ABA and MeJA signals, with 33 elements each (accounting for 73% of the total number of genes), indicating that most *PcCDPK* genes are involved in the response process of abscisic acid and methyl jasmonate. A total of 43 *PcCDPK* genes contain at least one stress response element in the promoter region, such as drought stress response element MBS, defense and stress response element TC-rich repeats, and low-temperature response element LTR. The above results indicate that *PcCDPK* genes may be involved in the growth and development of patchouli and its response to stress.

### 3.5 Expression of *PcCDPK* gene under continuous cropping and p-HBA treatment

**Figure 5:**
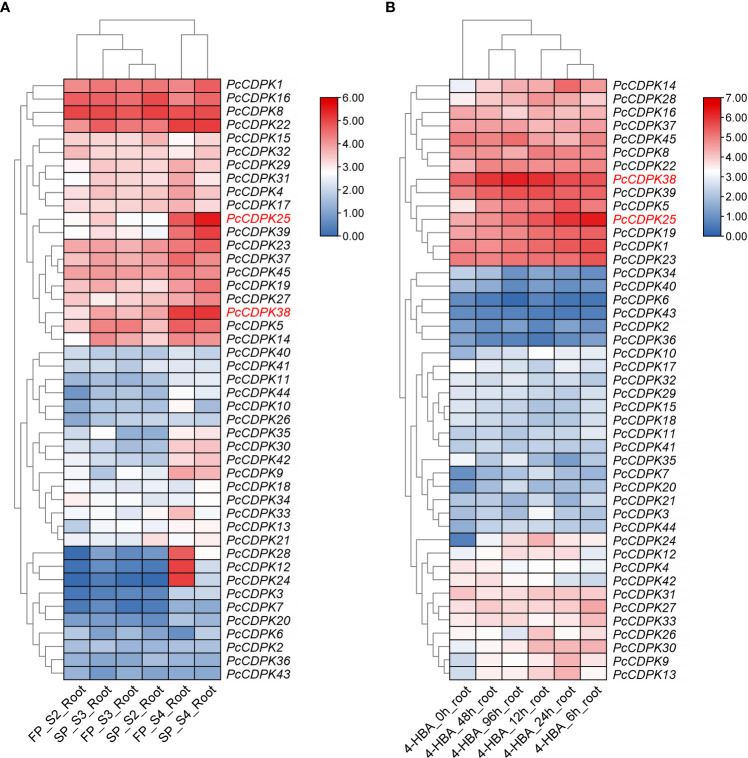
Expression profile of *PcCDPKs* in response to continuous cropping stress and p-HBA stress. **(A)** The expression level of *PcCDPKs* in the roots of patchouli in the first crop and continuous cropping for one year **(B)**. The expression level of *PcCDPKs* in the roots of patchouli under p-HBA stress treatment for 0 h, 6 h, 12 h, 24 h, 48 h, and 96 h.

Using transcriptome data from the roots of patchouli under continuous cropping stress and 1mmol/L p-hydroxybenzoic acid (p-HBA) stress, the expression of 45 *PcCDPK* gene family members under continuous cropping and allelochemicals stress was analyzed. The results are shown in **Figure 5**. The transcriptome data of the roots of patchouli from the first crop and continuous cropping for one year (**Figure 5A**) showed that *PcCDPK1*/*8*/*16/22* exhibited high expression levels during the S2, S3, and S4 growth stages of patchouli. The expression levels of genes such as *PcCDPK14/25/38/39* were relatively low in the early stages of growth, but significantly increased in the S4 stage. Moreover, compared to the first crop, these genes were significantly upregulated in the root system of continuous cropping for one year. It is speculated that these genes are highly likely to be key upregulated genes in response to continuous cropping stress. The three genes *PcCDPK12/24/28* were not expressed or had low expression levels in the S2 and S3 stages of patchouli growth, but their expression levels suddenly increased in the S4 stage of the first crop. However, the expression levels of these three genes in the S4 stage of continuous cropping were significantly lower than those in the S4 stage of the first crop, indicating that these three genes may be downregulated genes in response to continuous cropping stress.

### 3.6 Gene cloning, protein structure, and evolutionary analysis of *PcCDPK25* and *PcCDPK38*

**Figure 6:**
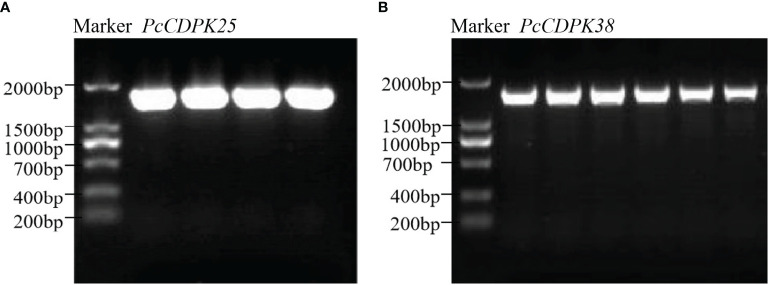
Bacterial solution PCR gel electrophoresis band of cloning *PcCDPK25/PcCDPK38.* **(A)** PCR gel electrophoresis band of *PcCDPK25*, **(B)** PCR gel electrophoresis band of *PcCDPK38*.

Using cDNA of the continuous cropping *P. cablin* root as the template, PCR amplification was carried out with clone primers, and the amplified product was subjected to agarose gel electrophoresis. Then, the gel cutting recovery product was constructed on a pBM16A vector through a Topost cloning kit, and the correct bacterial population sequencing was selected for the PCR band of the bacterial solution, and 1629 bp *PcCDPK25* fragment (**Figure 6A**) and 1716 bp *PcCDPK38* were obtained (**Figure 6B**, **Supplementary Table 2**).

**Figure 7:**
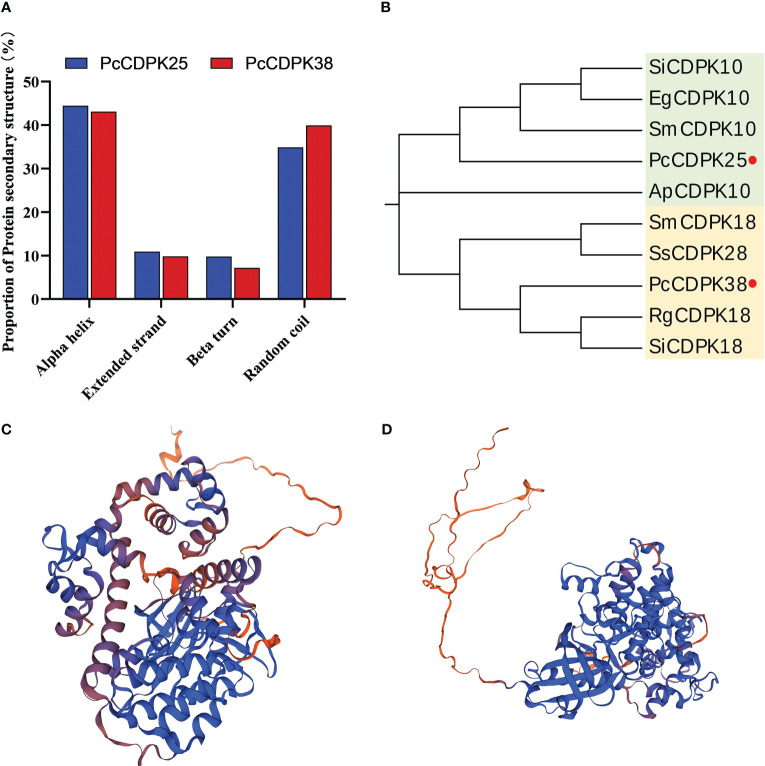
Analysis of the secondary and tertiary structures and phylogenetic evolution of PcCDPK25 and PcCDPK38 proteins. **(A)** The proportion of protein secondary structure of PcCDPK25 and PcCDPK38, **(B)** phylogenetic evolution analysis of PcCDPK25 and PcCDPK38, **(C)** the tertiary structure of PcCDPK25 protein, **(D)** the tertiary structure of PcCDPK38 protein.

Through the SOPMA online analysis website, the protein secondary structures of PcCPK25 and PcCPK38 were predicted. The results showed that the proportion of Alpha helix in PcCDPK25 and PcCDPK38 proteins was the highest, with 44.24% and 43.08% respectively, followed by Random coil with 36.01% and 39.93% respectively, and Extended strand with 11.33% and 9.81% respectively; The proportion of Beta turns is the lowest, only 8.41% and 7.18%, respectively (**Figure 7A**). The PcCDPK25 and PcCDPK38 proteins were compared and modelled with existing protein sequences in the Swiss Model database, and the tertiary structure of the PcCDPK25 and PcCDPK38 proteins was predicted as shown in **Figures 7C, D**.

Amino acid sequences for PcCDPK25 and PcCDPK38 were compared in the NCBI database, and a phylogenetic tree was constructed. This analysis incorporated CDPK sequences from various plant species including *Sesamum indicum* L., *Erythranthe guttata* (Fisch. ex DC.), *Andrographis paniculata* (Burm. f.), *Salvia miltiorrhiza* Bunge, *Rehmannia glutinosa* (Gaert.), and *Salvia splendens* Ker Gawl. These selected sequences displayed a high degree of similarity to the aforementioned two genes. The results showed that PcCDPK25 had a high similarity with SiCDPK10, EgCDPK10, and SmCDPK10, and had a sequence alignment consistency of 86.89%, 84.62%, 84.30%, and 83.58% with sesame, macadamia, *S. miltiorrhiza*, and *Andrographis paniculata*, respectively, indicating a close evolutionary phylogenetic relationship; PcCDPK38 has a close evolutionary relationship with RgCDPK18 and SiCDPK18, with base sequence consistency of 88.29%, 87.57%, 85.96%, and 84.99% with *R. glutinosa*, Sesame, *S. miltiorrhiza*, and *S. splendens*, respectively. The high sequence consistency indicates that *CDPKs* genes are highly conserved (**Figure 7B**, **Supplementary Figure S1**).

### 3.7 Subcellular localization of PcCDPK25 and PcCDPK38

**Figure 8:**
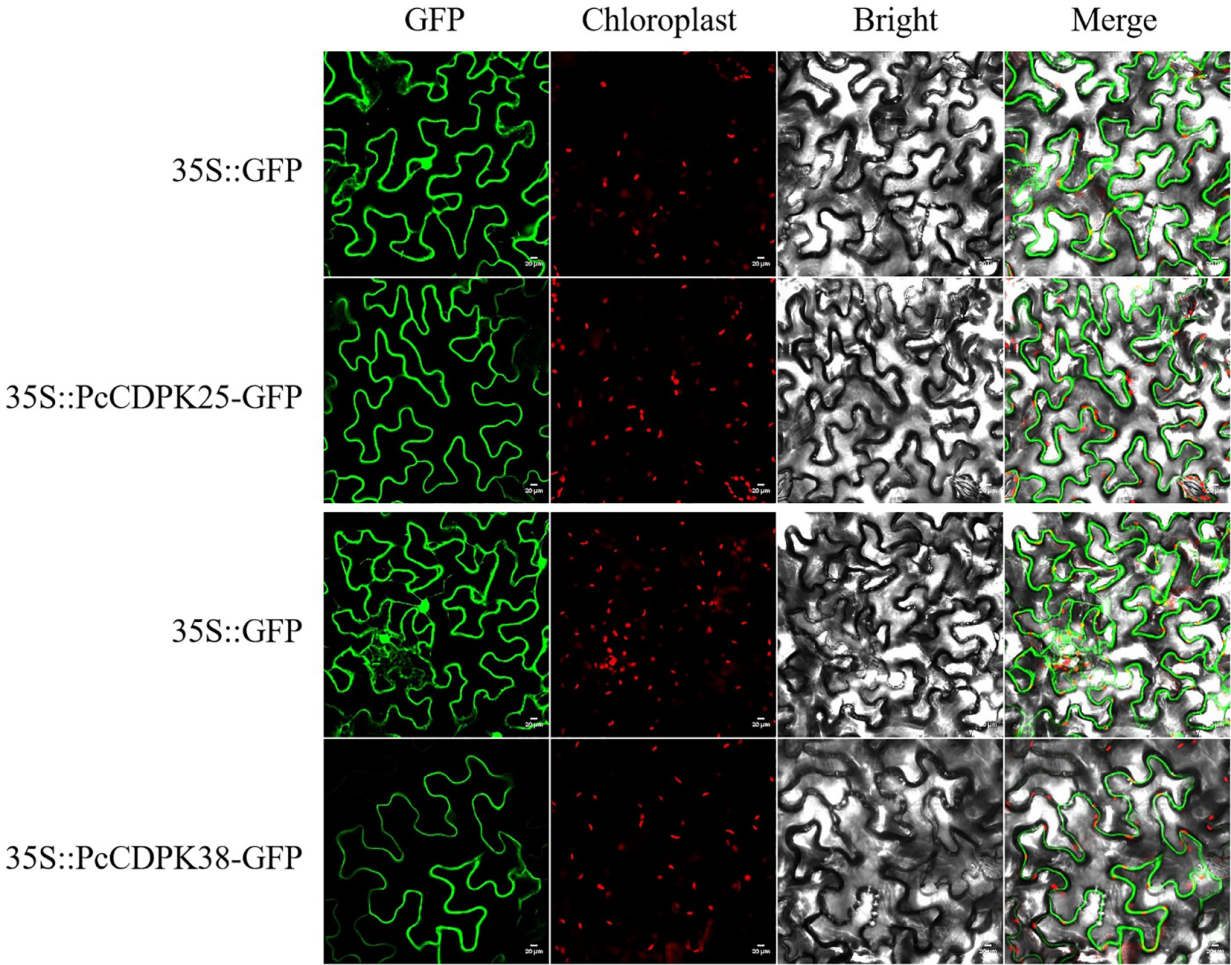
Subcellular localization of PcCDPK25 and PcCDPK38.

Plant overexpression vectors pBWA (V) HS-*PcCDPK25*-Glosgfp and pBWA (V) HS-*PcCDPK38*-GLosgfp were constructed using homologous arm primers for *PcCDPK25* and *PcCDPK38* by homologous recombination method. After sequencing the vector, it was confirmed that the target sequence was correct. Subsequently, Agrobacterium mediated transient expression technology was used to inject Agrobacterium GV3101 bacterial solution containing the fluorescent protein recombinant plasmid into tobacco cells for transient expression. Two days after injection, subcellular localization of PcCDPK25 and PcCDPK38 proteins was observed. The results indicated that the empty vector pBWA (V) HS-ccdb Glosgfp showed bright green fluorescence throughout the entire cell, while cells overexpressing *PcCDPK25* and *PcCDPK38* genes only showed bright green fluorescence in the plasma membrane (**Figure 8**), indicating that the vector constructed in this study can successfully express PcCDPK25 and PcCDPK38 proteins, both of which are localized in the plasma membrane.

### 3.8 The qRT-PCR analysis of *PcCDPK25* and *PcCDPK38*

**Figure 9:**
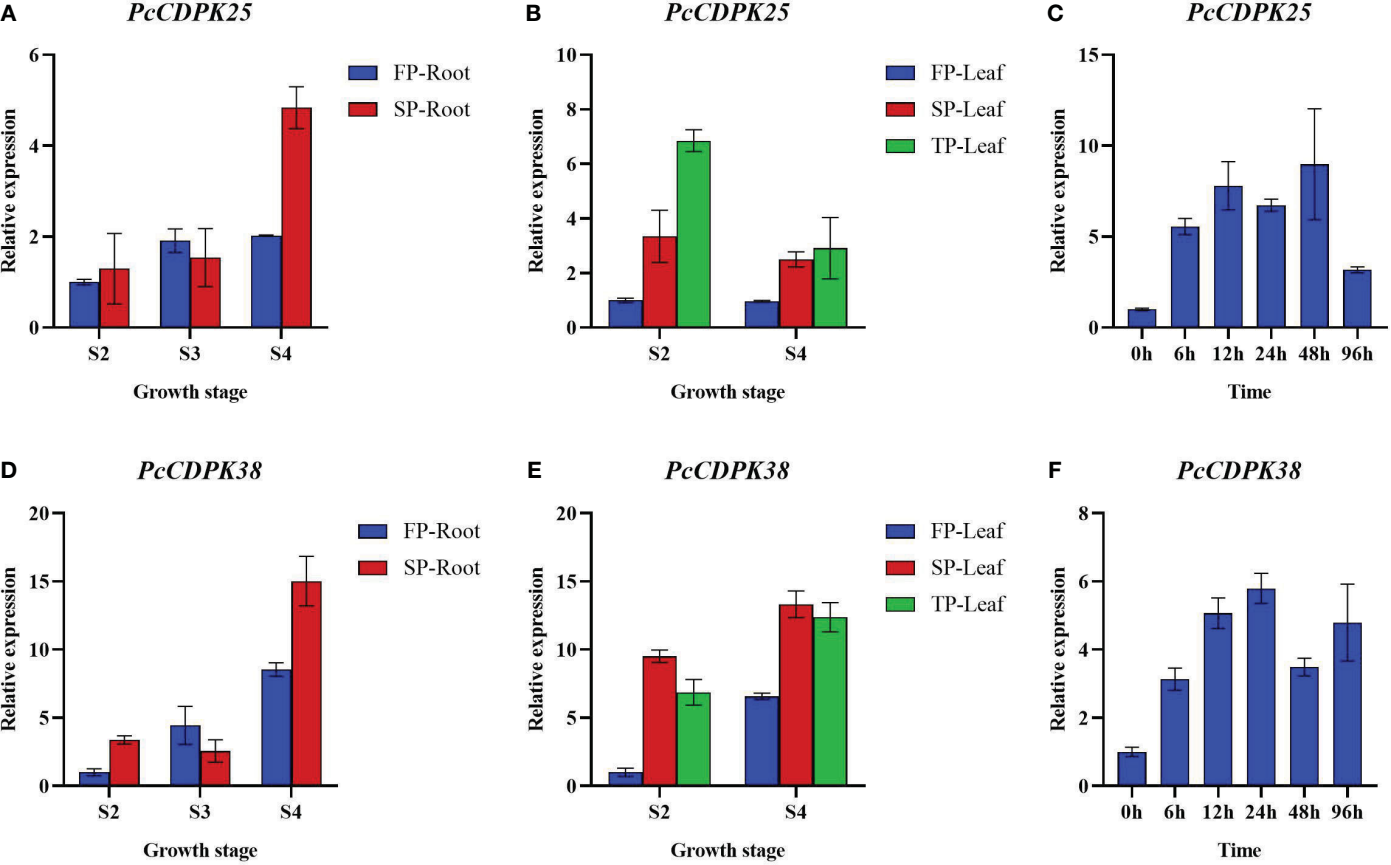
The expression of *PcCDPK25* and *PcCDPK38* genes at different growth stages of patchouli under continuous cropping stress and p-HBA stress.

To further reveal the expression patterns of two key *CDPK* genes under continuous cropping disorder and p-HBA stress, we examined the expression of *PcCDPK25* and *PcCDPK38* genes at different growth stages of patchouli under continuous cropping stress and p-HBA stress. The qRT-PCR results showed that as the root system of patchouli grew, the expression levels of *PcCDPK25* and *PcCDPK38* continued to increase, and the expression levels of the two genes under continuous cropping stress were significantly higher than the control during the S4 period of growth (**Figures 9A, D**); The expression level of *PcCDPK25* gene in the leaves of the S2 and S4 stages of patchouli is TP>SP>FP (TP; Third Panting, SP; Second Planting, FP; First Planting), indicating that the expression level of *PcCDPK25* is continuously increasing with the extension of continuous cropping years, while the expression level of *PcCDPK38* gene is SP>TP>FP, and the expression level of *PcCDPK38* gene is significantly higher in continuous cropping for one year and two years than in the first crop, indicating that continuous cropping significantly increases the expression level of *PcCDPK38* gene (**Figures 9B, E**); At 0 h, 6 h, 12 h, 24 h, 48 h, and 96 hours under p-HBA stress, the expression levels of *PcCDPK25* and *PcCDPK38* showed a general trend of first increasing and then decreasing with the prolongation of stress time, indicating that the expression of *PcCDPK25* and *PcCDPK38* genes was significantly affected by p-HBA stress (**Figures 9C, F**).

## 4 Discussion

*P. cablin* is one of the 20 common essential oil plants in the international market, and also an important raw material for dozens of traditional Chinese patent medicines and simple preparations, which makes it have important pharmaceutical and industrial values worldwide, and has a very broad application prospect (Singh et al., 2015). Continuous cropping obstacles have always been a bottleneck in the development of the patchouli planting industry, seriously hindering the standardized and industrialized cultivation of patchouli, and reducing the enthusiasm of pharmaceutical farmers for planting and regional economic development (Shen et al., 2021). The main causes of continuous cropping obstacles include soil nutrient deficiency and imbalance, self-toxicity of allelopathic substances, and aggravation of soil-borne diseases, which are the result of a combination of multiple factors in the “plant-soil-microbial” system (Guenzi and McCalla, 1996; Zeeshan Ul Haq et al., 2023). Using transcriptomics and metabolomics techniques, the mechanism of continuous cropping disorders has been studied in various medicinal plants, such as *Aconitum chuanxiong* (Liu et al., 2023), *P. cablin* (Yan et al., 2023), *Codonopsis pilosula* (Jiang et al., 2023), Ginseng (Shen et al., 2023), *Pinellia ternata* (He et al., 2022b), etc. Nonetheless, despite the extensive omics research, there remains a scarcity of comprehensive investigations into the functional roles of pivotal genes responsible for regulating continuous cropping, particularly within the context of continuous cropping disorders in patchouli. Consequently, it holds significant importance to delve into the molecular mechanisms governing the responses of target genes to continuous cropping disorders in patchouli. This study focused on the analysis of the *CDPK* gene family, which may play a role in regulating continuous cropping disorders in patchouli, drawing insights from a substantial pool of transcriptome data collected during the early stages of the research project. Through methods such as bioinformatics, gene cloning, subcellular localization, and qRT-PCR, the gene structure and function of gene family members were analyzed, revealing the transcription and expression patterns of genes, as well as the regulatory mechanisms under continuous cropping stress.

CDPK is widely involved in different biological processes of calcium signal transduction and can bind Ca^2+^ through the EF-hand domain and alter protein conformation in response to changes in the intracellular environment (Asano et al., 2012b). The number of *CDPK* genes varies greatly among different species, with 34, 31, 26, 40, and 18 *CDPK* genes found in Arabidopsis (Cheng et al., 2002), rice (Ray et al., 2007), potato (Fantino et al., 2017), corn (Zhao et al., 2021a), and melon (Zhang et al., 2017), respectively. This study screened 45 members of the *PcCDPK* gene family from the genomic data of patchouli and divided them into 4 CDPK subfamilies, Groups I-IV. Although the length and physicochemical properties of the encoded proteins vary among each member, they share similar genetic structures. 45 *PcCDPK* genes all contain one serine/threonine (Ser/Thr) kinase domain and four EF-hand domains, and *CDPK* genes in the same subfamily of the evolutionary tree have similar numbers of introns/exons and arrangement patterns, indicating that the genetic structure within the same subfamily is relatively more conservative during the evolutionary process. The *PcCDPK* members of the Group III subfamily, except for *PcCDPK40*, all contain the FRQ1 superfamily. However, *PcCDPK40*, as a member of the same subfamily, lacks this superfamily, which may be due to missing fragments during evolution or incomplete genome annotation information, and further exploration is needed. Genes with close branches in the phylogenetic tree often have similar gene structures and high homology. Therefore, *PcCDPKs* and their homologous genes *AtCDPKs* or *OsCDPKs* in the same branch may have similar functions. Huang (Huang et al., 2018) found that under drought and salt induced conditions, the expression level of *AtCPK1* in Arabidopsis significantly increased, suggesting that *PcCDPK14*, which has similar evolutionary development, may be involved in growth regulation under drought and salt stress. Corratge-Faillie (Corratge-Faillie et al., 2017) and Chen (Chen et al., 2019) demonstrated that AtCPK9 and AtCPK33 proteins are located on the cell membrane and highly expressed in guard cells, and mutant dysfunction exhibits a more sensitive phenotype to ABA regulation of stomatal movement but ion channel activity, while overexpression lines exhibit the opposite phenotype. Homologous to *AtCPK9* and *AtCPK33*, *PcCDPK11* may play a negative regulatory role in ABA signaling in the stomata of patchouli. Gene replication events play an important role in biological evolution (Leebens-Mack et al., 2019). Collinear analysis of species revealed 65 collinear gene pairs within the family, which may be due to species-specific genome-wide replication events during plant evolution that lead to an increase in gene family members.

An increasing body of research has consistently identified the involvement of *CDPKs* in modulating plant stress responses across diverse signaling pathways, including those associated with drought, cold, salinity, injury, and pathogen infection (Schulz et al., 2013; Kanchiswamy et al., 2014). *OsCPK12* can enhance the activity of antioxidant enzymes in rice, reduce the accumulation of ROS, and enhance salt tolerance (Asano et al., 2012b); Overexpression of *OsCPK13* significantly increased the low-temperature resistance of transgenic rice lines (Saijo et al., 2010). Corn can induce high expression of *ZmCPK1* under low temperature stress, while inhibiting the expression of *ZmCPK25*, improving its cold resistance (Berberich and Kusano, 1997; Weckwerth et al., 2015). Within this study, it was established that 43 *PcCDPK* genes concurrently possess at least one hormone-responsive element and one stress-responsive element. This discovery further solidifies the significance of these gene family members in governing the growth, development, and external stress responses of patchouli. Under continuous cropping stress and allelochemicals p-HBA stress, the expression level of *PcCDPK1/5/8/22/16/25/38/39* gene was higher, while the expression level of *PcCDPK2/6/36/40/43* gene was lower. Among these genes, the expression levels of *PcCDPK25* and *PcCDPK38* displayed significant upregulation. Furthermore, qRT-PCR results indicated an upward trend in the expression of the *PcCDPK25*/*38* genes as patchouli grew under continuous cropping conditions. Interestingly, under p-HBA stress, their expression levels initially increased and then decreased, suggesting that these two genes likely serve as key players in responding to the stress induced by continuous cropping. In order to elucidate their functions, we cloned the *PcCDPK25*/*38* sequences, which share close similarities with the calcium signaling system-related genes *RgCDPK10*/*18* known to respond to continuous cropping in *R. glutinosa* plant (Yang et al., 2017b). Utilizing the fusion expression method of *CDPK* with green fluorescent protein, we observed that the majority of Arabidopsis CDPK proteins predominantly localized to the plasma membrane (Dammann et al., 2003). Subcellular localization prediction indicates that PcCDPK25/38 protein containing soybean acylation and palmitoylation sites are all located on the plasma membrane on the localization of CDPKs in alfalfa, indicating that the N-terminal phosphorylation site may play a key role in the subcellular localization of PcCDPK protein (Zhao et al., 2021b). The *OsCDPK18*, which is homologous to *PcCDPK38* in rice, can synergistically regulate defense related genes with *MPK5*, while differentially regulating developmental related genes; *OsCDPK9*, homologous to *PcCDPK25*, enhances drought stress tolerance by enhancing stomatal closure and enhancing plant osmoregulation ability, while homologous Arabidopsis *CPK10* and *CPK30* participate in signal transduction of ABA and abiotic stresses (Zou et al., 2010; Wei et al., 2014; Li et al., 2020).

In summary, 45 *CDPK* gene family members were identified from patchouli, distributed on 28 chromosomes and playing important roles in stress. The *PcCDPK25* and *PcCDPK38* genes are subcellular located on the plasma membrane and are likely key positive regulatory factors involved in continuous cropping stress and allelochemical stress in patchouli, making them candidate genes for subsequent functional analysis. The researchers used VIGS technology to silence the *CsARR-9* gene in cucumbers and found that silencing this gene can promote cell viability, enhance thylakoid accumulation, and increase chloroplast storage, thereby improving the photosynthetic efficiency of cucumbers and reducing the self-toxicity generated during cucumber continuous cropping (Bu et al., 2023). In future research, we will further characterize the functions of the *PcCDPK25* and *PcCDPK38* genes in patchouli, and use techniques such as gene editing, stable overexpression, and virus silencing to alter the functional status of the genes, thereby alleviating the harm caused by continuous cropping of patchouli. This study lays the research foundation for in-depth analysis of the function of patchouli CDPK and its molecular mechanism in response to the continuous cropping of patchouli.

## 5 Conclusions

In summary, this study recognized 45 members of the *PcCDPK* gene family in the entire genome of patchouli and divided them into four subfamilies. The physicochemical properties, phylogeny, collinearity, gene structure and cis-acting elements were analyzed by bioinformatics. Using transcriptome data to analyze the expression of continuous cropping stress in each member, we identified two key genes, *PcCDPK25* and *PcCDPK38*. Cloning of *PcCDPK25* and *PcCDPK38* genes resulted in 1629 bp and 1716 bp long fragments, respectively. The subcellular localization findings indicated that both PcCDPK25 and PcCDPK38 proteins were located on the plasma membrane. Additionally, the qRT-PCR results demonstrated an upregulation in the expression levels of these genes when patchouli was subjected to continuous cropping stress, underscoring their significant roles in responding to this specific stress condition in patchouli. These outcomes serve as a foundational framework for delving deeper into the functional aspects of *PcCDPK* genes in patchouli under continuous cropping obstacles.

## Data availability statement

The original contributions presented in the study are included in the article/**Supplementary Material**. Further inquiries can be directed to the corresponding author.

## Author contributions

XL: Conceptualization, Data curation, Formal Analysis, Investigation, Methodology, Resources, Software, Validation, Visualization, Writing – original draft, Writing – review & editing. MZ: Conceptualization, Data curation, Investigation, Methodology, Software, Validation, Visualization, Writing – original draft, Writing – review & editing. JY: Data curation, Formal Analysis, Investigation, Software, Supervision, Visualization, Writing – review & editing. YL: Conceptualization, Formal Analysis, Methodology, Resources, Supervision, Visualization, Writing – original draft, Writing – review & editing. HY: Conceptualization, Formal Analysis, Methodology, Resources, Software, Writing – review & editing. HC: Data curation, Formal Analysis, Investigation, Resources, Validation, Visualization, Writing – review & editing. DY: Data curation, Methodology, Resources, Software, Validation, Writing – review & editing. YW: Conceptualization, Funding acquisition, Investigation, Project administration, Supervision, Writing – original draft, Writing – review & editing.
