# Supplementary material for: Identification of the CDPK gene family in patchouli and functional analysis in response to continuous cropping stress
Source: Front Plant Sci. 2023 Nov 22;14:1300073. doi: 10.3389/fpls.2023.1300073 (PMC10702526; doi:10.3389/fpls.2023.1300073)
Supplement: Supplementary file 1 [file DataSheet_1.docx]

Supplementary Material


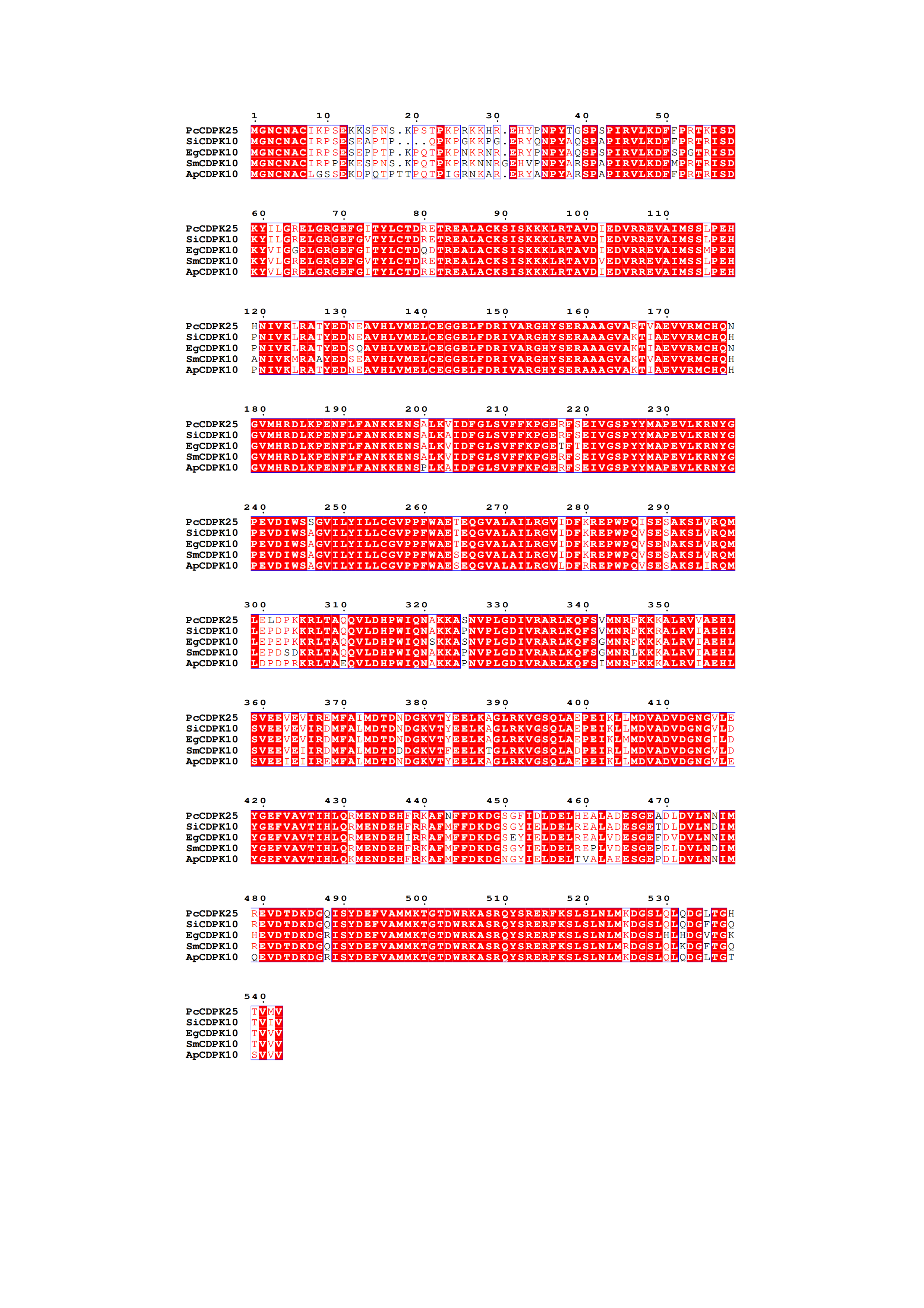


Supplementary Figure 1. Multiple sequence alignment of PcCDPK25 with other species.


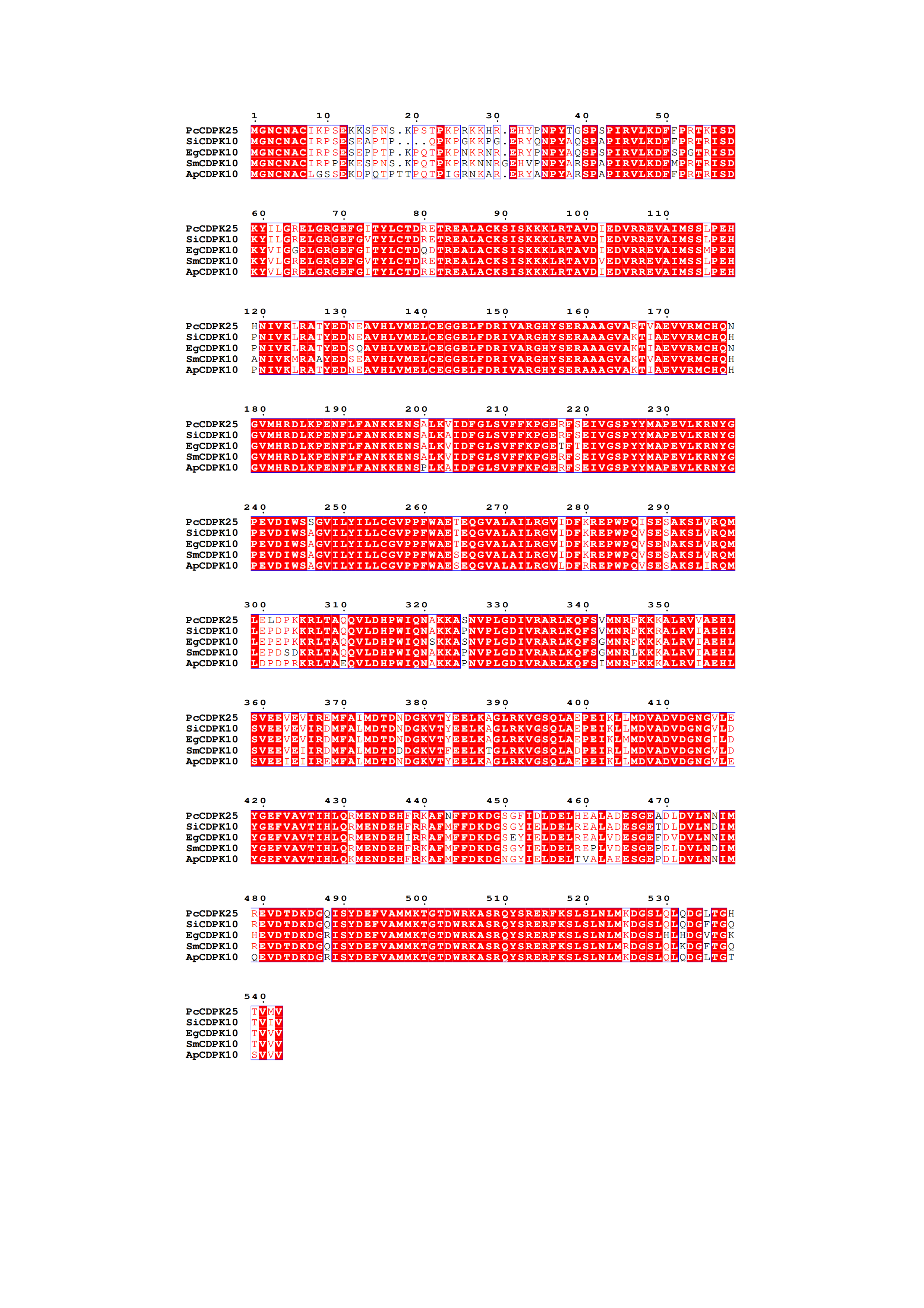


Supplementary Figure 2. Multiple sequence alignment of PcCDPK38 with other species.
